# The contents of the potentially harmful elements in the arable soils of southern Poland, with the assessment of ecological and health risks: a case study

**DOI:** 10.1007/s10653-019-00372-w

**Published:** 2019-07-19

**Authors:** Agnieszka Gruszecka-Kosowska, Agnieszka Baran, Magdalena Wdowin, Katarzyna Mazur-Kajta, Tomasz Czech

**Affiliations:** 1 Department of Environmental Protection, Faculty of Geology, Geophysics, and Environmental Protection, AGH University of Science and Technology, al. Mickiewicza 30, 30-059 Kraków, Poland; 2 Department of Agricultural and Environmental Chemistry, Faculty of Agriculture and Economics, University of Agriculture in Krakow, al. Mickiewicza 21, 31-120 Kraków, Poland; 3 Mineral and Energy Economy Research Institute, Polish Academy of Sciences, ul. J. Wybickiego 7A, 31-261 Kraków, Poland; 4 Department of International Economic Relations, Faculty of Economics and Management, Opole University of Technology, ul. Prószkowska 76, 45-758 Opole, Poland

**Keywords:** Agricultural soils, Trace elements, Contamination factors, Ecological risk, Human health risk assessment, Bioavailability

## Abstract

Agricultural soil samples were collected from the areas where edible plants had been cultivated in southern Poland. The PHE content decreased in proportion to the median value specified in brackets (mg/kg d.m.) as follows: Zn (192) > Pb (47.1) > Cr (19.6) > Cu (18.8) > Ni (9.91) > As (5.73) > Co (4.63) > Sb (0.85) > Tl (0.04) > Cd (0.03) > Hg (0.001) > Se (< LOQ). No PHE concentrations exceeded the permissible levels defined in the Polish law. The PHE solubility (extracted with CaCl_2_) in the total concentration ranged in the following order: Fe (3.3%) > Cd (2.50%) > Ni (0.75%) > Zn (0.48%) > Cu (0.19%) > Pb (0.10%) > Cr (0.03%). The soil contamination indices revealed moderate contamination with Zn, ranging from uncontaminated to moderately contaminated with Pb, and, practically, no contamination with other PHEs was identified. The ecological risk indices revealed that soils ranged from uncontaminated to slightly contaminated with Zn, Pb, As, Cu, and Ni. The PCA indicated natural sources of origin of Co, Cu, Hg, Sb, Zn, Cr, and Pb, as well as anthropogenic sources of origin of Cd, Ni, As, and Tl. The human health risk assessment (HHRA) for adults and children decreased in the following order of exposure pathways: ingestion > dermal contact > inhalation of soil particles. The total carcinogenic risk values for both adults and children were at the acceptable level under residential (1.62E−05 and 6.39E−05) and recreational scenario (5.41E−06 and 2.46E−05), respectively, as well as for adults in agricultural scenario (1.45E−05). The total non-carcinogenic risk values for both adults and children under residential scenario (1.63E−01 and 4.55E−01, respectively), under recreational scenario (2.88E−01 and 6.69E−01, respectively) and for adults (1.03E−01) under agricultural scenario indicated that adverse health effects were not likely to be observed. Investigated soils were fully suitable for edible plant cultivation.

**Electronic supplementary material:**

The online version of this article (10.1007/s10653-019-00372-w) contains supplementary material, which is available to authorized users.

## Introduction

Arable land is of special concern since, due to its special designation for food production, it needs to meet strictly defined requirements, in terms of contaminant content. Primarily, those contaminates can penetrate soil directly, as a result of either natural or anthropogenic activities, but also in the process of self-cleaning of other environmental components, especially air. When the environment becomes more and more polluted, it seems to be inevitable that the concentrations of adverse substances in the soils used for food production may also increase. Moreover, while the areas of arable land are often surrounded by those of intense industrial activity and/or agglomerations with intense traffic and household contaminants, taking care of the arable land quality becomes the matter of highest importance.

Metals and metalloids, often jointly called the ‘heavy metals’, belong to the most significant inorganic soil contaminants. Some of the PHEs (As, Cd, Cr, Co, Ni, Pb) are classified as possibly carcinogenic to humans (IARC 2012) by the International Agency for Research on Cancer (IARC). On the other hand, spectrum of potentially toxic effects of heavy metals is very wide, from fatigue and headache (Pratush et al. 2018) to a series of symptoms associated with cardiovascular, renal, blood, nervous, and bone diseases (Zhuang et al. 2009) to severe damage of such critical organs as kidneys or the nervous system (Wadhwa et al. 2015; Cabral-Pinto et al. 2018).

Agricultural soils are threatened by the PHE pollution due to rapidly expanding urban and industrial areas or the intensive use of fertilizers and agrochemicals for the crop protection purposes (Kelepertzis et al. 2015; Klimek-Kopyra et al. 2015; Šukalić et al. 2018). Recent research on agricultural soils revealed the accelerated accumulation of heavy metals and associated health risk, especially due to greenhouse agricultural cultivation (Wan et al. 2019) and the usage of fertilizers and fungicides (Huang et al. 2019). Moreover, high impact of PHEs contamination on soils via atmospheric deposition is mainly generated by coal combustion (Peng et al. 2019) that in Poland is very common. Exceedance of regulation limits concerning PHEs content was observed in soils that are adjacent to megalopolises, large motorways and industrial enterprises (Barsova et al. 2019). The total PHE content is an important indicator of soil contamination, although this measure is not sufficient to assess any adverse effects that have actually occurred in the soil ecosystem (Baran et al. 2014, 2018; Kim et al. 2015; Jiang et al. 2019). In risk assessment, the data about the content of soluble or exchangeable trace element forms are useful since such elements display mobility from the solid phase to soil solution, once they become bioavailable (Jiang et al. 2019). PHEs do not accumulate in soils only, but they can also migrate to groundwater (Satapathy and Panda 2018) or penetrate higher links of the trophic chain (Dziubanek et al. 2015, 2017). What is more, PHEs are non-biodegradable and they can migrate from soil to groundwater causing further contamination. The speciation research of Wang et al. (2019) indicated that PHEs were very mobile and bioavailable in studied agricultural soils.

What is more and more popular is the trend of the so-called healthy eating (Kapetanaki et al. 2014; Banna et al. 2016; Rafacz 2019). That trend is mostly associated with the consumption of fresh food products, i.e., vegetables and fruit, grown by local farmers, without artificial fertilizers or plant protection chemicals. The local produce grown in such conditions is sold regionally, often brought straight from the farms to “fresh food markets”, without washing with chemicals or packaging in plastic foil or containers. Also in Poland, such a trend becomes quite popular, which is especially visible in the southern regions of the country where industrial and post-industrial lands are mixed with farming regions. One can expect that the tendency will spread among the population. It is worth noting that arable lands constitute 73% of farmlands in Poland, and, together with forests, represent 90% of the country’s surface area (GUS 2016). Chemical monitoring of arable soils is conducted by the National Environmental Monitoring System maintained by the IUNG Institute of Soil Science and Plant Cultivation in Puławy. The system produces 216 measurement-control profiles for arable soils in the whole country. The measurements have been taken every 5 years since 1995 (GIOŚ 2017).

Taking above under consideration, it could be assumed that PHEs concentrations in arable soils and consequently in edible plants will increase affecting human health. Thus, investigations of the arable soils of southern Poland, where edible plants (i.e., vegetables, fruit, and cereals) were simultaneously cultivated, were the goal of the study. The detailed objectives of the research were to determine (1) the mineralogical and geochemical characteristics of arable soils in southern Poland, (2) the total concentration of selected PHEs (As, Cd, Co, Cr, Cu, Hg, Ni, Pb, Sb, Se, Tl, and Zn), with their bioavailability for plants, (3) the values of contamination indices for the total PHE content, and (4) the ecological and human health risk assessment values resulting from the presence of PHEs in arable soils.

## Materials and methods

### Study area, soil sampling, and sample preparation

**Fig. 1:**
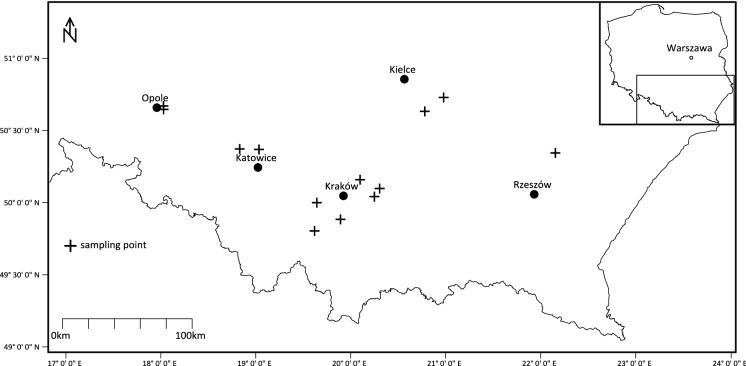
Agricultural soil sampling site locations in southern Poland

The study areas were selected in southern Poland. Taking the administrative division of the country into consideration, 12 soil samples were collected in the Małopolskie Region, 4 in the Opolskie, 4 in the Śląskie, 6 in the Świętokrzyskie, and 4 in the Podkarpackie (Fig. 1). The soil sampling sites were located in the areas where edible plants (vegetables, fruit, and cereals) had been cultivated and subsequently sold to the residents of nearby cities on the local fruit and vegetable markets.

A total of 30 soil samples were collected from the depth of 0–25 cm each in 2015 and 2016, in accordance with the PN-ISO 10381-4:^2007^ Polish Standard protocol. The coordinates of the sample locations were recorded, using a portable Global Positioning System (GPS). About 1 kg of each soil sample was collected from a 1 × 1 m square; each sample consisted of five subsamples (taken from the square corners and the diagonal intersection) of each sample grid. For each soil sample, duplicates were collected and analyzed in the same way as the basic samples. In the region of southern Poland where the investigations were conducted, soils were classified as cambisols, podzols, and luvisols according to World Reference Base for Soil Resources (IUSS Working Group WRB 2015). The soil samples were air-dried at room temperature (25 °C) and homogenized, with mass reduction by the ring and cone method. Then, the soil samples were passed through a 2-mm sieve to remove plant parts and gravel material before further analysis.

### Sample analysis

Active (pH_H2O_) and potential (pH_KCl_) pH values were determined in duplicate soil samples using 5 g of soil sample and 25 cm^3^ of deionized water and 1 M KCl, respectively (PN-ISO 10390:^1997^ protocol). The organic carbon (*C*_org_) content was determined by the Tiurin method (PN-ISO 14235:^2003^). Approximately 0.2 g of soil was weighed into 100 cm^3^ conical flasks, 10 cm^3^ of potassium dichromate in sulfuric acid (VI) at a concentration of 0.06 mol/dm^−3^ and about 0.2 g of HgSO_4_ were added. Analysis of organic carbon content was performed in each soil sample in duplicate. The prepared sample in conical flasks with a glass funnel as a reflux condenser was heated on a hot plate for 2 h, and after cooling, three drops of phenolphthalein were added. The solution thus prepared was titrated with Mohr’s salt to change the color from orange to green to dark red. The organic matter (OM) content was determined gravimetrically on duplicate 5 g oven-dried samples, in a furnace, kept at 450 °C for 16 h (ISO 10694:^1995^).

X-ray diffraction (XRD) patterns were recorded for powdered soil samples, with the use of a Rigaku MiniFlex diffractometer (CuKα radiation), in the range of 1°–14°2*θ*, with the 0.05°2*θ* step. The values of interplanar distances, obtained from the X-ray images (in angstroms = 0.1 nm), were used to identify the mineral phases that were part of the samples being studied. For the diffraction data processing, a ClayLAB, ver. 1.0, was used. The identification of mineral phases was carried out, based on a PCPDFWIN, ver. 1.30, formalized by JCPDS-ICDD.

A FEI Quanta 200 FEG scanning electron microscope (SEM), equipped with an EDS EDAX micro-area analysis system, as well as a backscattered electron detector (YAG BSE), was also used in the research. Samples were sprayed with carbon before observation.

Thermal analysis (DTA/TG) was performed, using an STA 449 F3 Jupiter Netzsch apparatus, coupled with a quadrupole mass spectrometer, QMS 403C Aeolos, and an FTIR Bruker spectrometer.

The total PHE concentrations (As, Cd, Co, Cr, Cu, Hg, Ni, Pb, Sb, Se, Tl, and Zn), as well as those of Al and Fe extracted in 3 g of soil samples with 28 cm^3^ of *aqua regia* (HNO_3_ and HCl at 3:1 *v*/*v*) after mineralization in 105 °C for 2 h, were determined by inductively coupled plasma mass spectrometry ICP-MS (ELAN 6100; PerkinElmer, Waltham, MA, USA) according to the USEPA 6020B protocol (USEPA 1998) and the ISO protocol (PN-EN ISO 17294-2:^2006^).

The bioavailable forms of PHEs were extracted, 2 g of each soil sample, with 20 cm^3^ of 0.01 mol CaCl_2_/dm^3^ (Rauret 1998; Pueyo et al. 2004; Wieczorek et al. 2018) and shaken for 2 h. After extraction, the samples were centrifuged at 3000 rpm for 10 min. The supernatants were separated from the precipitates through a filter. The contents of the bioavailable forms of PHEs were determined, using the ICP-OES (inductively coupled plasma optical emission spectroscopy) method, with an Optima 7300 DV (PerkinElmer).

### Quality control

Soil analyses were performed, with the observance of the certified standard analytical quality control procedure (PN-EN ISO 17294-1:^2007^). To achieve impartial and unequivocal ICP-MS results, the elements were also measured, using ICP-OES inductively coupled plasma optical emission spectroscopy (OPTIMA 7300DV; PerkinElmer, Waltham, MA, USA), in accordance with the USEPA 6020B protocol (USEPA 1998) and the ISO protocol (PN-EN ISO 11885:^2009^). Certified reference material (CRM) (soil ERM^®^-CC141) was analyzed at the same time. The recovery from the CRM soil was between 81% and 112% for the majority of the analyzed PHEs, except for As, Co, Cr, Ni. Reagent blanks and duplicates of each three samples were used to ensure quality assurance and quality control. All the reagents used in the laboratory analysis were analytically pure. The results of the samples were within the allowable error change values. Analytical bias was statistically insignificant (*p* = 0.05). The accuracy of the ICP-MS and ICP-OES systems was satisfactory and verified by six different solution injections. As an internal standard, Rh was used. For the ICP-MS analysis to minimize the impact of interferences, the element correction equations were used for each element.

The limit of quantification (LOQ) values of the investigated PHEs were as follows: Al < 0.005 mg/dm^3^, As < 0.001 mg/dm^3^, Cd < 0.003 mg/dm^3^, Co < 0.0002 mg/dm^3^, Cr < 0.005 mg/dm^3^, Cu < 0.001 mg/dm^3^, Fe < 0.02 mg/dm^3^, Hg < 0.0001 mg/dm^3^, Ni < 0.001 mg/dm^3^, Pb < 0.0001 mg/dm^3^, Sb < 0.0002 mg/dm^3^ Se < 0.01 mg/dm^3^, Tl < 0.0001 mg/dm^3^, and Zn < 0.001 mg/dm^3^.

### Statistical analysis

The statistical analysis involved the determination of mean, median, standard deviation, minimum, maximum, and coefficient of variation (CV%). The relationships between the parameters in soil samples were assessed, using the Pearson correlation coefficients and PCA. Statistical analyses were performed, using the Microsoft Excel 2007 spreadsheet and the Statistica 12 package.

### Soil quality and contamination indices

In order to indicate a potential contamination of analyzed agricultural soils by PHEs under investigation, the permissible levels of metals and metalloids were used (Regulation of the Polish Minister of Environment on how to conduct pollution assessment the surface of the earth of 1 September 2016 (RMŚ 2016). Since not all the elements were included in the above-mentioned Polish Regulation, the Canadian soil quality guidelines for the protection of environmental and human health were also applied (CCME 2007). Furthermore, although the total concentration of metals and metalloids is still the most popular chemical indicator, contamination indices were also calculated as the first step of determining a potential grade of soil contamination. The pollution indices, based on the total PHEs concentration, were divided into two groups: individual and complex. The individual pollution indices determine the soil contamination separately for each element. The following single soil contamination indices were selected for our investigations: geoaccumulation index (*I*_geo_), enrichment factor (EF), contamination factor (CF), single pollution index (PI), threshold pollution index (PI_T_), and potential contamination index (PCI). However, while PHEs occur in the environment as mixtures, complex contamination indices determining the impact of many pollutants seem to be more adequate. The following complex soil contamination indices were chosen for the investigations: contamination degree (C_deg_), modified contamination degree (mC_deg_), sum of pollution index (PI_sum_), sum of threshold pollution index (PI_Tsum_), average pollution index (PI_Avg_), average pollution threshold index (PI_TAvg_), pollution load index (PLI), and improved Nemerow pollution index (PI_N_). A brief description of the soil contamination indices, used in this study, is given in ESM_1. The local geochemical background B_*n*_ values (Kabata-Pendias 2011), needed for calculations, were used in the *I*_geo_ index, as well as those from Rudnick and Gao (2014), determining concentration in the upper continental crust as preindustrial values. Fe as a heavy metal, characterized by a low variability of occurrence, was chosen for the calculations of the EF factor (Kowalska et al. 2018). Calculations were performed twice; first, the B_Fe_ concentration was chosen from crust soils (Kabata-Pendias 2011), and then as a regional geochemical background for loamy soils in Poland (Kabata-Pendias and Pendias 1999; Kabata-Pendias 2011). In the CF factor, significant preindustrial concentration values of PHEs were taken from the upper continental crust, as reference values, in accordance with Rudnick and Gao (2014), while in the single PI index, the background values for the PHEs data from Kabata-Pendias and Pendias (1999) and Kabata-Pendias (2011) were used. In the case of the single PI index and the potential contamination PCI index, the local values of geochemical background were used (Kabata-Pendias 2011). In the case of the single PI_T_ index, the Polish soil environment quality standards were used (RMŚ 2016). Whenever the PHEs under examination were missing in the Polish Regulation, the values from the Canadian soil quality guidelines for the protection of environmental and human health (CCME 2007) were applied.

### Ecological risk indices

To determine a more realistic impact of elements on living organisms, several ecological risk indices were developed. They were based on the threshold effect concentrations, below which harmful effects were unlikely to be observed (TECs) and on probable effects of the concentrations (PECs), above which harmful effects were likely to be observed (MacDonald et al. 2000). Thus, in the second step of determining soil quality, some sediment quality indices were also used for soils (Qui 2010; Inengite et al. 2015; Weissmanová and Pavlovský 2017). The following single ecological risk indices were selected for the investigations: potential ecological risk coefficient (Er), hazard quotient (HQ), and modified hazard quotient (mHQ); the complex ecological risk indices were the following: potential ecological risk index (RI), hazard index (HI), mean probable effects level quotient (mPELq), mean effect range median quotient (mERMq), ecological contamination index (ECI), and contamination severity index (CSI). A brief description of the ecological risk indices, used in the study, is given in ESM_2. The above values of individual PHE permissible levels, specified in RMŚ (2016) and CCME (2007), were used as standard.

### Human health risk assessment

The methodology used for human health risk assessment (HHRA) was based on the guidelines provided by the US Environmental Protection Agency (USEPA 1989). In the research, three site-specific exposure scenarios were investigated. The first exposure scenario was assumed to be agricultural: soil cultivation by adults was investigated. The second exposure scenario was assumed to be residential and the third one recreational. Within the second and the third exposure scenarios, the populations of both adults and children were investigated. Moreover, under each exposure scenario, three exposure routes were investigated: inhalation of resuspended soil particles, accidental soil ingestion, and dermal contact with soil. Thus, average daily doses (ADD) for ingestion exposure and dermal exposure, as well as exposure concentrations (EC) for the inhalation route, were calculated according to Eqs. (1)–(3) (USEPA 2001, 2004, 2009; Izquierdo et al. 2015; Wcisło et al. 2016), respectively:

1 $${\text{ADD}}_{{{\text{soil}}\;{\text{ingestion}}}} = \left( {C_{\text{soil}} \times {\text{CF}} \times {\text{IR}}_{\text{ing}} \times {\text{FI}} \times {\text{EF}} \times {\text{ED}} \times {\text{RBA}}} \right)/\left( {{\text{BW}} \times {\text{AT}}} \right)$$

2 $${\text{ADD}}_{{{\text{dermal}}\;{\text{contact}}}} = \left( {C_{\text{soil}} \times {\text{CF}} \times {\text{AF}} \times {\text{ABS}}_{\text{d}} \times {\text{EF}} \times {\text{ED}} \times {\text{EV}} \times {\text{SA}}} \right)/\left( {{\text{BW}} \times {\text{AT}}} \right)$$

3 $${\text{EC}} = \left( {C_{\text{soil}} \times {\text{ET}} \times {\text{EF}} \times {\text{ED}}} \right)/\left( {{\text{PEF}} \times {\text{AT}}} \right)$$

where ADD is the average daily dose (mg/kg day); EC, exposure concentration (mg/m^3^); *C*_soil_, mean concentration of analyzed PHE in soil samples (mg/kg); CF, unit conversion factor (10^−6^ kg/mg); IR_ing_, soil ingestion rate (mg/kg); FI, fraction ingested from contaminated source (unitless); EF, exposure frequency (days/year); ED, exposure duration (years); RBA, relative bioavailability factor (unitless); BW, body weight (kg); AF, adherence factor of soil to skin (mg/cm^2^-event); ABS_d_, dermal absorption factor (unitless); EV, event frequency (events/day); SA, skin surface area available for contact (cm^2^); ET, exposure time for receptor (h/day); PEF, soil-to-air particulate emission factor (m^3^/kg); AT, averaging time (ED × 365 days for non-carcinogens and 70 × 365 days for carcinogens; in days for ingestion and dermal contact; in hours for inhalational exposure). The exposure parameters, used for the HHRA calculations under concerned site-specific scenarios, are given in Table 1.

**Table 1:** Exposure parameters used for the HHRA calculations in the study

Exposure parameters	Residential		Recreational		Agricultural	References
	Adult	Child	Adult	Child	Adult	
IR_ing_—soil ingestion rate (mg/kg)	100	200	100	200	100	USEPA (2011)
IR_inh_—inhalation rate for receptor (m^3^/h)	0.83	0.31	0.83	0.31	0.83	USEPA (2002, 2008)
CF—unit conversion factor (kg/mg)	10^−6^	10^−6^	10^−6^	10^−6^	10^−6^	USEPA (2011)
FI—fraction ingested from contaminated source (unitless)	1	1	1	1	1	USEPA (2001)
ET—exposure time for receptor (h/day)	24	24	4	4	8	Site specific
ED—exposure duration (years)	24	6	24	6	40	US EPA (2001) and Wcisło et al. (2016)
EF—exposure frequency (days/year)	365	365	96^a^	96^a^	250^b^	USEPA (2011); site specific; Journal of Laws (1951)
EV—event frequency (events/day)	1	1	1	1	1	Site specific
AF—adherence factor of soil to skin (mg/cm^2^-event)	0.07	0.2	0.07	0.2	0.07	USEPA (2011)
SA—skin surface area available for contact (cm^2^)	6032	2373	6032	2373	3527	USEPA (2014)
PEF—soil-to-air particulate emission factor (m^3^/kg)	1.36 × 10^9^	1.36 × 10^9^	1.36 × 10^9^	1.36 × 10^9^	1.36 × 10^9^	USEPA (2002)
BW—body weight (kg)	70	15	70	15	70	USEPA (2011)
AT—averaging time non-carcinogens (days)	8760	2190	8760	2190	14,600	USEPA (2001)
AT—averaging time carcinogens (days)	25,550	25,550	25,550	25,550	25,550	USEPA (2001)
AT—averaging time non-carcinogens (hours)	210,240	52,560	210,240	52,560	210,240	USEPA (2009)
AT—averaging time carcinogens (hours)	613,200	613,200	613,200	613,200	613,200	USEPA (2009)

^a^Site specific: 96 days = 8 non-winter months × 4 weeks × 3 days

^b^Site specific, based on the Polish Act on non-working days of 18 January 1951 (Journal of Laws 1951)

To determine the non-carcinogenic and carcinogenic risks arising from the content of PHEs analyzed in soils, the HQ and CR values were calculated, respectively. The values of the hazard indices (HQ) in oral and dermal exposure pathways were calculated, using Eq. (4), and those regarding the inhalational route, using Eq. (5). The values of the carcinogenic risks (CR) for oral and dermal exposure pathways were calculated according to Eq. (6) and those regarding the inhalational exposure, using Eq. (7) (USEPA 1989, 2009; Wcisło et al. 2016):

4 $${\text{HQ}} = {\text{ADD}}/{\text{RfD}}_{\text{o}}$$

5 $${\text{HQ}} = {\text{EC}}/{\text{RfC}}$$

6 $${\text{CR}} = {\text{ADD}} \times {\text{SF}}_{\text{o}}$$

7 $${\text{CR}} = {\text{EC}} \times {\text{IUR}}$$

where HQ is the hazard quotient (unitless); CR, carcinogenic risk (unitless); ADD, average daily dose (mg/kg day); EC, exposure concentration (mg/m^3^); RfD_o_, oral reference dose (mg/kg day); RfC, reference concentration (mg/m^3^); SF_o_, oral slope factor; IUR, inhalation unit risk (mg/m^3^). The reference doses (RfD_d_) and slope factor values (SF_d_) for dermal contact were calculated according to Eqs. (8) and (9), respectively (Wcisło et al. 2016):

8 $${\text{RfD}}_{\text{d}} = {\text{RfD}}_{\text{o}} /{\text{GIABS}}$$

9 $${\text{SF}}_{\text{d}} = {\text{SF}}_{\text{o}} \times {\text{GIABS}}$$

where GIABS is the fraction of contaminant absorbed in the gastrointestinal tract (unitless).

The total non-carcinogenic risk of PHEs was determined by the hazard index (HI_*t*_) values, according to Eq. (10), and the total carcinogenic risks (CR_*t*_) of analyzed PHEs according to Eq. (11):

10 $${\text{HI}}_{t} = {\text{HQ}}_{1} + {\text{HQ}}_{2} + \cdots + {\text{HQ}}_{i}$$

11 $${\text{CR}}_{t} = {\text{CR}}_{1} + {\text{CR}}_{2} + \cdots + {\text{CR}}_{i}$$

where 1 − *n* were individual PHEs.

The values of toxicological parameters, used for the calculations of the site-specific HHRA, are given in Table 2.

**Table 2:** Toxicological parameters used for the HHRA calculations in the study

Toxicological parameters	RfD_o_^a^	SF_o_^a^	RfC^a^	IUR^a^	RBA^a^	ABS_d_^b^	GIABS^c^
	mg/kg day	(mg/kg day)^−1^	mg/m^3^	(mg/m^3^)^−1^	unitless	unitless	unitless
As	3.00E−04	1.50E+00	1.50E−05	4.30E−06	0.6	0.03	1
Cd	1.00E−03	–	1.00E−05	1.80E−06	1	0.01	0.025
Co	3.00E−04	–	6.00E−06	9.00E−06	1	0.01	1
Cr (III)	1.50E+00	–	–	–	1	0.01	0.013
Cr (VI)	3.00E−03	5.00E−01	1.00E−04	8.40E−05	1	0.01	0.025
Cu	4.00E−02	–	–	–	1	0.01	1
Hg	3.00E−04	–	3.00E−04	–	1	0.01	0.07
Ni	2.00E−02	–	9.00E−05	2.60E−07	1	0.01	0.04
Pb	1.50E−03^d^	8.50E−03	–	–	1	0.01	1
Sb	4.00E−04	–	–	–	1	0.01	0.15
Se	5.00E−03	–	2.00E−02	–	1	0.01	0.3
Tl	1.00E−05	–	–	–	1	0.01	1
Zn	3.00E−01	–	–	–	1	0.01	1

RfD_o_, oral reference dose; SF_o_, oral slope factor; –, not available

^a^USEPA (2019)

^b^USEPA (2018)

^c^USEPA (2004)

^d^EFSA (2010)

The target risk value was set to be equal to 1 (HQ = 1) for both non-carcinogenic risk, for each PHE investigated (individual HQ values), and the total non-carcinogenic risk, defined by HI_t_ values. As to carcinogenic risk, the acceptable risk level was set to be equal to 1 × 10^−6^ for an individual PHE and equal to 1 × 10^−4^ for the sum of carcinogenic PHEs (USEPA 1989, 1991).

## Results

### Physicochemical characterization of soils

The mean total concentrations of the PHEs, analyzed in the agricultural soils of southern Poland, the pH values, the contents of organic matter and organic carbon, and the content of the < 0.02 mm fraction are presented in Table 3. Soil pH was in the range of 4.8–7.2 for pH_H2O_ and 3.9–7.5 for pH_KCl_; the median value of pH indicated that soils were from neutral to slightly acidic. Fraction < 0.02 mm was in the range of 7–50%, with the mean value of 26% (GIOŚ 2017). The content of organic matter differed from 1.01 to 6.00%, with the mean value of 2.73%. The *C*_org_ content varied from 5.95 to 34.8 g/kg, and the median value was equal to 15.2 g/kg. Based on the fraction < 0.02 mm content, the investigated soils were generally classified as loamy. The concentration of elements, characterized by a low variability of occurrence (As and Fe), ranged from 0.25 to 1.16%, with the mean concentration of 0.65% in the case of Al and in the range 0.34–3.23%, with the mean content of 0.98%, in the case of Fe. Based on twelve investigated PHEs, the Se concentrations were < LOQ in all the investigated samples. As to Cd, Hg, and Tl, the minimum concentrations were < LOQ and the maximum ones were equal to 3.53 mg/kg d.m. for Cd, 0.52 mg/kg d.m. for Hg, and 0.35 mg/kg d.m. for Tl. Their median concentrations were equal to 0.03 mg/kg, 0.001 mg/kg, and 0.04 mg/kg, respectively. The other PHEs were ordered decreasingly as follows, with the median values (mg/kg d.m.) given in brackets: Zn (192) > Pb (47.1) > Cr (19.6) > Cu (18.8) > Ni (9.91) > As (5.73) > Co (4.63) > Sb (0.85).

**Table 3:** Total PHE concentrations and the physicochemical parameters of the agricultural soils in southern Poland

Parameters	Units	Soil (*n* = 30)				Permissible concentration, Polish guidelines (RMŚ 2016)	Canadian Soil Quality Guidelines for the Protection of Environment and Human Health (CCME 2007)		
		Min	Max	Mean	Median		Agricultural	Residential	Commercial
pH_H2O_	–	4.8	7.9	6.5	6.8	–	–	–	–
pH_KCl_	–	3.9	7.5	6.0	6.3	–	–	–	–
Organic matter	%	1.01	6.00	2.73	2.39	–	–	–	–
*C*_org_	g/kg	5.88	34.8	15.8	13.9	–	–	–	–
Al	%	0.25	1.16	0.65	0.56	–	–	–	–
Fe	%	0.34	3.23	0.98	0.85	–	–	–	–
As	mg/kg	1.40	16.6	6.64	5.73	20	12	12	12
Cd		< LOQ	3.53	0.39	0.03	3	1.4	10	22
Co		1.44	12.3	4.92	4.63	30	40	50	300
Cr		2.08	35.4	18.8	19.6	300	64	64	87
Cu		5.23	110	26.6	18.8	150	63	63	91
Hg		< LOQ	0.52	0.05	0.001	4	6.6	6.6	24
Ni		1.06	27.1	11.5	9.91	150	45	45	89
Pb		14.2	263	63.8	47.1	250	70	140	260
Sb		0.03	3.07	1.23	0.95	–	20	20	40
Se		< LOQ	< LOQ	< LOQ	< LOQ	–	1	1	2.9
Tl		< LOQ	0.35	0.10	0.04	–	1	1	1
Zn		38.5	1224	283	192	500	250	250	410

< LOQ, below the limit of quantification; –, not applicable

RMŚ (2016) Regulation of the Polish Minister of Environment on how to conduct pollution assessment the surface of the earth of 1 Sept. 2016. OJ 2016. item 1395

CCME (2007) Canadian Council of Ministers of the Environment. 2007. Canadian soil quality guidelines for the protection of environmental and human health: Summary Tables. Updated September. 2007. In: Canadian environmental quality guidelines. 1999. Canadian Council of Ministers of the Environment. Winnipeg.

### Mineralogical characterization of soils

**Fig. 2:**
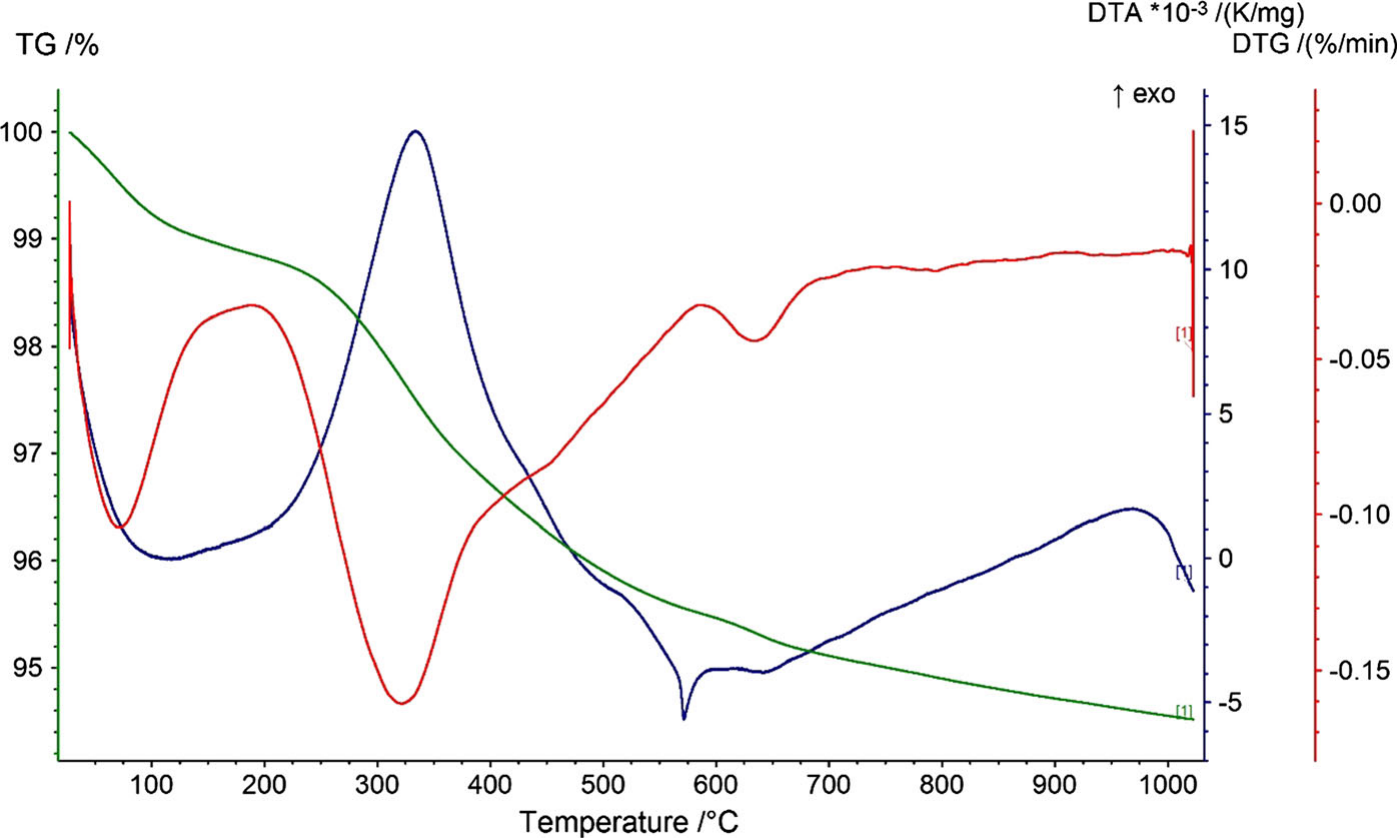
TG analyses of the agricultural soils samples

**Fig. 3:**
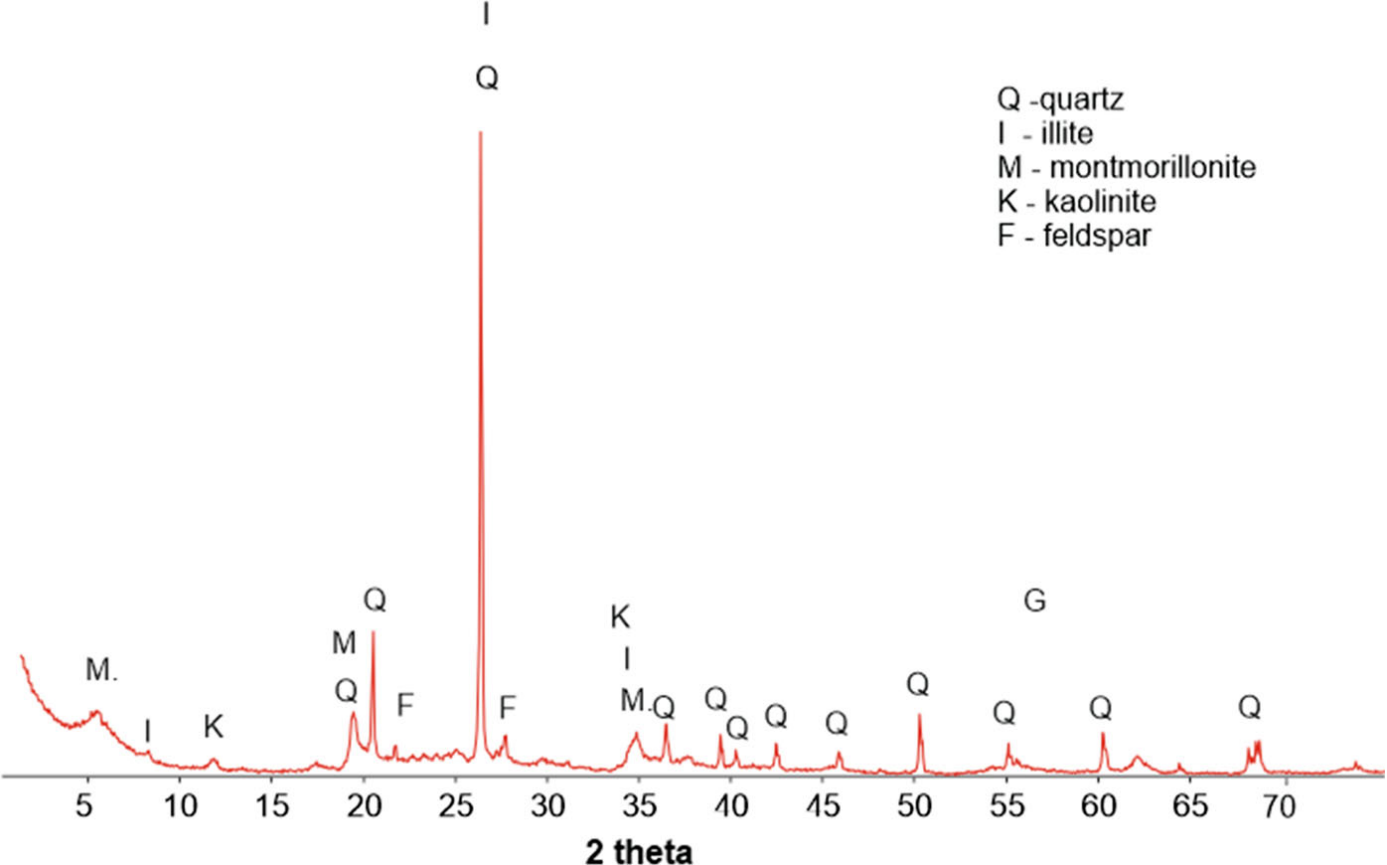
An example of the XRD diffraction pattern of the agricultural soil samples

**Fig. 4:**
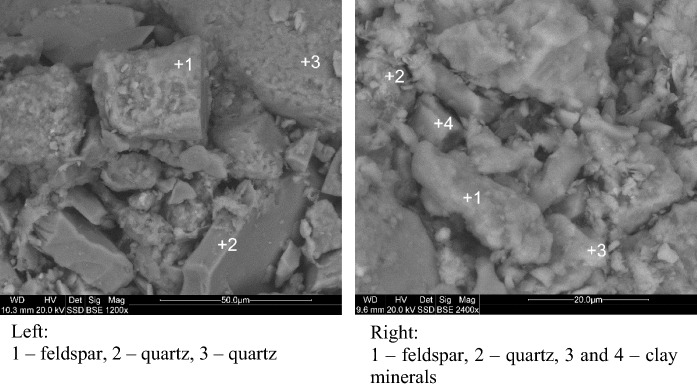
SEM microphotographs of the agricultural soil samples, with EDS analysis in microareas

The TG analysis (Fig. 2) showed loss in weight, in the proportion of only about 5%, which suggested a low content of organic matter and a small amount of water in soil. The fastest loss was observed in the temperature range of 0–600 °C. The DTA curve indicated two main endothermic reaction bands, the first one in the range of 100–200 °C, associated with the removal of water, and the second one at 570 °C, associated with the transition of quartz from a low-temperature quartz form (quartz-α) to a high-temperature one (quartz-β). In addition, two important exothermic reaction bands were visible: the first one, in the range of 250–400 °C, associated with the dehydration and thermal decomposition of humic substance in a mineralized residue, consisted mainly of clay minerals and oxides, as well as carbonates formed during the decomposition of organic matter (de Oliviera et al. 2009), and the second one, in the range of 900–1000 °C, associated with the material decomposition and the formation of new mineral phases. The mineralogical XRD analysis (Fig. 3) revealed that the mineral composition of the analyzed soil samples was dominated by quartz. In addition, small amounts of feldspars, illite, montmorillonite, kaolinite, and goethite were visible. The samples differed only in the amount of individual components. Based on that, one might conclude that the clay minerals and organic matter, confirmed by the TGA analysis, were responsible for the presence of PHEs in soils. The morphology investigations and grain observations, using SEM–EDS, indicated that quartz with various crystal sizes was a dominant component (Fig. 4). Moreover, small aggregates of clay minerals were observed, together with feldspar crystals. Due to a very similar mineral composition of all the investigated soils, the highest concentrations of PHEs could be associated with anthropogenic activities rather than mineral composition.

### Soil quality standards

The mean concentrations of twelve PHEs analyzed were compared to the permissible concentrations of metals and metalloids, specified in RMŚ (2016) for Ground Type II-2, in reference to the content of the < 0.02 mm fraction and the pH_KCl_ values (Table 3). Neither PHE concentration exceeded the permissible levels defined in RMŚ (2016). As to Sb, Se, and Tl, for which no permissible levels were determined in the Polish law, the values specified in the Canadian soil quality guidelines for the protection of environment and human health (CCME 2007) were adopted, and the guideline concentrations were not exceeded either. However, we need to mention that the Canadian soil guidelines show much lower permissible levels than the Polish ones. Since the Canadian guidelines were developed for the protection of environment and human health, the mean and median values of the other PHEs investigated in the soils of southern Poland were also compared to them. Similarly, none of the PHEs exceeded the guideline values; however, the Zn and Pb levels were exceeded depending on the fact whether the mean or median values were taken into consideration.

### Assessment of the potential bioavailable forms of PHEs

The content of bioavailable forms of PHEs in soil samples varied, within a wide range, from <  LOQ to 88.1 mg/kg d.m (Table 4). The highest contents of bioavailable forms of PHE_S_ were determined for Zn, followed by Fe > Ni > Pb > Cd > Cu > Cr. However, it was found that PHE solubility in 0.01 mol CaCl_2_/dm^3^ was low in the studied soils and ranged from 0.10 to 3.30%, in respect of their total content. PHE solubility ranged in the following order: Fe (3.3%) > Cd (2.50%) > Ni (0.75%) > Zn (0.48%) > Cu (0.19%) > Pb (0.10%) > Cr (0.03%) of the total concentration of those elements. Similarly, low solubility of trace elements was found in the studies of Li et al. (2014) and Kelepertzis et al. (2015), which highlighted the low extraction efficiency of the CaCl_2_ extraction, especially in the pH range characterizing the soils under consideration. The results confirmed a higher mobility of Cd in comparison with that of other trace elements. Many authors revealed that cadmium was very mobile and highly toxic for living organisms even at low concentrations (An 2004; Wieczorek et al. 2018). Moreover, Kim et al. (2015) suggested that the content of trace elements in soils, extracted with 0.01 mol CaCl_2_/dm^3^, was well correlated with the response of living organisms. The studies by Zhang et al. (2010) indicated higher values of the correlation coefficients between the CaCl_2_-soluble fractions of soil and the contents of four of the investigated metals (Cd, Cu, Zn, and Pb) in plants. As a three-step process, bioavailability included environmental availability (availability of elements in soil); environmental bioavailability (uptake of elements by organisms); and toxicological bioavailability (negative influence of elements on organisms) (Kim et al. 2015). In the context of the described project, the bioavailability of elements was primarily influenced by soil properties. The behavior of trace elements in soils was controlled by many factors such as: pH, redox condition, electric conductivity, organic matter content, or granulometric composition (Shaheen and Rinklebe 2014; Baran et al. 2018; Jiang et al. 2019). Generally, it was found that solubility of trace elements increased at low soil pH and decreased in soils with high organic carbon content. The examined soils showed slightly acidic reaction and average organic carbon content. Those factors could have influenced a lower mobility of the analyzed PHEs.

**Table 4:** PHE forms that are bioavailable for plants in the agricultural soils in southern Poland (extracted with CaCl_2_)

PHEs (mg/kg)	Soil (*n* = 30)				% of mean total concentration
	Min	Max	Mean	Median	
Cd	0.0005	0.25	0.06	0.03	15.4
Cr	< LOQ	0.04	0.005	–	0.03
Cu	< LOQ	0.68	0.05	0.01	0.19
Fe	0.1	4.0	0.5	0.3	0.01
Ni	< LOQ	1.37	0.11	0.05	0.96
Pb	0.02	0.53	0.07	0.04	0.11
Zn	0.02	4.30	1.37	1.12	0.48

< LOQ, below the limit of quantification; –, not applicable

### Soil contamination indices

For the soil quality determination, single and complex indices were calculated, based on the total concentrations of PHEs (Table 5). The values of the geoaccumulation index (*I*_geo local_), together with the local values of geochemical background for southern Poland (Kabata-Pendias and Pendias 1999; Kabata-Pendias 2011), indicated pollution Class 0 for As, Cd, Cr, Cu, Hg, Ni, Sb, Se, and Tl (practically uncontaminated), Class I for Pb (uncontaminated to moderately contaminated), and Class II for Zn (moderately contaminated). When the PHE concentrations in the upper continental crust (Rudnick and Gao 2014) were used as background values Class 0 stayed for As, Co, Cr, Cu, Hg, Ni, Se, and Tl, although Class II (moderately contaminated) was determined for Cd, Pb, Sb, and Zn. The calculations of the enrichment factor (EF), with Fe concentrations in crust soils as reference element (Kabata-Pendias 2011), indicated a severe enrichment in case of Zn, Pb, and Se, moderately severe enrichment of As, Cd, and Cu, moderate enrichment of Hg, minor enrichment of Co, Cr, Ni, and Sb, and no enrichment of Tl. When Fe contents in the loamy soils of Poland (Kabata-Pendias and Pendias 1999) were used as reference, the PHE concentrations in soil demonstrated moderately severe enrichment of Zn, moderate enrichment of Pb and Se, minor enrichment of As, Cd, Cu, and Hg, and no enrichment of Co, Cr, Ni, Sb, and Tl. The contamination factor (CF) values indicated a considerable contamination with Pb and Zn; moderate contamination with As, Cd, Cu, Hg, and Sb; and low contamination with Co, Cr, Ni, Se, and Tl. The values of the single pollution index (PI) indicated a strong pollution of soils with Zn, moderate pollution with As, Cd, Cu, Pb, and Se, and lack of pollution with Co, Cr, Hg, Ni, Sb, and Tl. Using the threshold values (RMŚ 2016; CCME 2007) in the calculation of the PI_T_ index, the values for each PHE indicated no pollution. The potential contamination index (PCI), calculated with the analyzed maximum concentrations of PHEs in soils, defined a low contamination with Cr and Tl, moderate contamination with Co, Ni, and Sb, and severe contamination with As, Cd, Cu, Hg, Pb, Se, and Zn.

**Table 5:** Contamination and ecological risk indices of the agricultural soils of southern Poland

PHEs	Soil (*n* = 30)											
	*I*_geo local_	*I*_geo crust_	EF_Fe crust_	EF_Fe loam_	CF	PI	PI_T_	PCI	C_deg_	mC_deg_	PI_sum_	PI_Tsum_
As	0	0	6	2	1	1.5	0.3	3.7	21	1.7	17.4	2
Cd	0	II	6	2	4	1.4	0.1	13				
Co	0	0	3	< 1	< 1	< 1	0.2	1.5				
Cr	0	0	2	< 1	< 1	< 1	0.1	0.9				
Cu	0	0	6	2	1	1.4	0.2	6.0				
Hg	0	0	4	2	1	< 1	0.0	9.0				
Ni	0	0	2	< 1	< 1	< 1	0.1	1.1				
Pb	I	II	11	4	4	2.6	0.3	11				
Sb	0	II	3	1	3	< 1	0.1	1.7				
Se	0	0	13	4	1	3.0	0.1	3.0				
Tl	0	0	< 1	< 1	< 1	< 1	0.1	0.4				
Zn	II	II	19	6	4	4.4	0.6	19				

PHEs	Soil (*n* = 30)												
	PI_Avg_	PI_TAvg_	PLI	PI_N_	Er	HQ	mHQ	RI	HI	mPELq	mERMq	ECI	CSI
As	1.4	0.2	0.9	0.4	13.8	0.33	0.86	237	2.0	0.36	0.27	2.07	0.28
Cd					130	0.13	0.40						
Co					1.42	0.16	–						
Cr					0.41	0.06	0.44						
Cu					4.75	0.18	0.56						
Hg					40.0	0.01	0.21						
Ni					1.22	0.08	0.56						
Pb					18.8	0.26	1.39						
Sb					21.5	0.06	–						
Se					–	0.08	–						
Tl					1.11	0.01	–						
Zn					4.22	0.57	1.77						

*I*_geo_, geoaccumulation index; EF, enrichment factor; CF, contamination factor; PI, single pollution index; PI_T_, single threshold pollution index; PCI, potential contamination index; C_deg_, contamination degree; mC_deg_, modified contamination degree; PI_sum_, sum of the pollution index; PI_Tsum_, sum of the threshold pollution index; PI_Avg_, average pollution index; PI_TAvg_, average threshold pollution index; PLI, pollution load index; PI_N_, improved Nemerow pollution index; Er, single index of the ecological risk factor; HQ, hazard quotient; mHQ, modified hazard quotient; RI, potential ecological risk index; HI, hazard index; mPELq, mean probable effect level quotient; mERMq, mean effect range median quotient; ECI, ecological contamination index; CSI, contamination severity index; –, not applicable

When describing the complex contamination indices, it was observed that the contamination degree (C_deg_) showed a considerable proportion of the investigated soil contamination, while the modified contamination degree (mC_deg_) denoted a low degree of contamination, the average pollution index (PI_Avg_) indicated a good quality of soil, the integrated pollution index (IPI) revealed moderate pollution, the pollution load index (PLI) showed no pollution, and the improved Nemerow pollution index (PI_N_) indicated Grade 1 of soil (safety domain). For PI_sum_ and PI_Tsum_, the calculated index values were equal to 12.3 and 2.8, respectively.

Both single and complex soil contamination indices varied depending on the background values applied (Štrbac et al. 2017). Classifications indicated the lowest soil contamination when threshold values were used as a geochemical background, lower when the background values, being specific for the country or even the region, were used, and finally the highest when general-background values were used, i.e., those referring to the continental Earth’s crust. Since Zn, Pb, and Cu ores are mined and processed, together with the accompanying elements in southern Poland, and rock outcrops also occur on the land surface, the local background values seem to be the most appropriate to apply. Thus, it can be summarized that the *I*_geo_ classification indicated a moderate contamination of soils by Zn, uncontaminated to moderate contamination by Pb, and practically no contamination by other investigated PHEs in southern Poland. The EF factor revealed a moderate severe enrichment of soils with Zn, moderate enrichment with Pb and Se, minor enrichment with As, Cd, Cu, and Hg, and no enrichment with Co, Cr, Ni, Sb, or Tl. The CF factor indicated a considerable contamination by Pb and Zn, moderate contamination by As, Cd, Cu, Hg, and Sb, and low contamination by Co, Cr, Ni, Se, and Tl. The PI index indicated a strong pollution by Zn, moderate pollution by As, Cd, Cu, Pb, and Se, and lack of pollution by Co, Cr, Hg, Ni, Sb, and Tl. Finally, the PI_T_ index indicated no pollution by each of the investigated PHEs.

### Ecological risk

To determine soil quality for the purpose of edible plant cultivation, single and complex ecological risk indices were calculated (Table 5). The potential ecological risk coefficient (Er) indicated a considerable potential ecological risk of Cd, moderate potential ecological risk of Hg, and low potential ecological risks of As, Co, Cr, Cu, Ni, Pb, Sb, Tl, and Zn. For Se the Er value was not determined due to the lack of toxic-response factor value. The hazard quotient (HQ) values denoted potential hazards in the cases of As, Cd, Co, Cr, Cu, Pb, Tl, and Zn, although no adverse effects were determined for Hg, Ni, Sb, and Se.

The potential ecological risk indices (RI) of the analyzed PHEs indicated a moderate ecological risk. The hazard index (HI) denoted a moderate hazard from the concentrations of investigated PHEs in soils. The mHQ indices revealed a moderate severity of contamination by Zn, low severity of contamination by Pb, very low severity of contamination by As, Cu, and Ni, and none to very low severity of contamination by Cd, Cr, or Hg. For Co, Sb, Se, and Tl, the mHQ values were not determined due to the lack of TEL, PEL, and SEL values in the literature. The mean probable effect level quotient (mPELq) of the investigated PHEs indicated a medium–low degree of contamination, with the probability of being toxic equal to 8%, while the mean effect range median quotient (mERMq) pointed at a medium–low priority site, with the probability of being toxic equal to 9%. The ecological contamination index (ECI) revealed that the investigated soils were at the border between uncontaminated and uncontaminated to slightly contaminated (the calculated ECI value: 2.07). Based on their percentage contributions to ECI, the PHE values were ordered in the following decreasing sequence: Zn > Pb > As > Cu > Ni > Cr > Cd > Hg. Finally, the contamination severity index (CSI) value equal to 0.28 indicated that the studied soils of the southern Poland were uncontaminated by the PHEs under consideration.

### Correlation and PCA

The above-described soil quality and ecological indicators were confirmed by the results of the correlation analysis (Table 6). In the respective studies, pH showed no significant correlation with the contents of all the elements. The element content was significantly either positively (Al, Fe, Co, Cu, Cr, Sb, and Zn) or negatively (Ni) correlated with the clay content. A high positive correlation between the elements and the clay content might suggest that the clay fraction played a dominant role in the mobility and sorption of the analyzed elements. Moreover, a lot of significant positive correlations between the elements and clay might point at the natural sources of PHE soils. Besides, a significantly positive correlation between the Pb content and *C*_org_ was observed. It is widely known that a significant positive correlation between the elements indicates that they have the same sources and display a similar behavior during transport (Baran and Wieczorek 2015). A lot of positively correlated pairs of elements were observed (Table 6). However, negative correlations were observed in Cd, Ni, As, and Tl in the studied soils. The negative correlations between some individual pairs of trace elements indicated that they might have come from various sources, and were usually associated with anthropogenic origin (Baran and Wieczorek 2015; Wieczorek et al. 2018). While analyzing the relationship between the contents of individual elements on the one hand and Fe and Al on the other hand, the latter were treated separately, while the positive correlation between the Al and Fe contents and the investigated PHEs suggested the elements’ natural content in the soil. The positive correlations between Fe and Al on the one hand and Co, Cu, Hg, Sb, Zn, Cr, and Pb on the other hand were indicated in the studies that confirmed the natural origin of the elements (Table 6). The negative correlations between the Fe and Al contents and the Cd, Ni, As, and Tl ones might point at anthropogenic origin of the investigated PHEs. However, it should be noted that the correlations obtained for the majority of relations were either very low, low, or medium. The principal component analysis (PCA) confirmed the above observations and extracted two principal components (PCs), explaining 44.88% of the total variance of the dataset. PC1, explaining 26.21% of the total variance, had a significant positive loading on clay, Al, Fe, Co, and Sb. Those results revealed that PC1, covering the PHE contents in the soil, had natural sources. Moreover, the high loading for clay and Fe suggested the importance of those minerals in binding trace element ions in the studied soils. PC2 explained 18.66% of the total variance, with a strong positive loading on As, Ni, Cd, Tl (Table 7). The combination of trace elements in PC2 suggested their anthropogenic origin and sources. For example, Ni and Cd have been commonly used in industry and their high quantities can be found on the areas surrounding developed urban areas. Moreover, the high Ni content in the environment is an important indicator of recent anthropogenic pollution (de Castro-Català et al. 2016). The described studies were conducted in southern Poland, in the region characterized by a large diversity of geological structures and agricultural and industrial development, as well as anthropogenic pressures. Southern Poland is the area of intense coal mining and processing in the Silesia (Szczepańska and Twardowska 1999; Jończy and Gawor 2017), mining and processing of Zn and Pb ores in the Olkusz area (Krzaklewski et al. 2004; Postawa and Motyka 2019), and of Cu ores in the Lower Silesia (Potysz et al. 2018). Besides the urban agglomerations of Kraków and Wrocław, the Upper and Lower Silesia Regions are also located in southern Poland, with significant contamination of the environment due to coal-burning heating plant operations, as well as wood and waste burning in poor quality stoves (Kobza et al. 2018), not to mention huge local traffic (Adamiec 2017). Atmospheric deposition of heavy metals in Poland is one of the highest in Europe, with the value of 68.2 mg/m^2^/year (Shi et al. 2018). Also, intense animal and vegetable production introduces PHEs to soils (Dach and Starmans 2005). On the other hand, southern Poland is also the land where the most productive soils occur in the so-called loess belt spreading from Ukraine to Germany across southern Poland (Labaz et al. 2019).

**Table 6:** Relationships between the soil sample properties

*n* = 30	pH	clay	*C*_org_	Al	Fe	As	Cd	Co	Cr	Cu	Hg	Ni	Pb	Sb	Tl
Clay	0.07														
*C*_org_	0.07	0.01													
Al	0.12	**0.55**	–												
Fe	0.15	**0.64**	0.07	**0.79**											
As	0.23	–	0.12	–	–										
Cd	0.25	–	0.06	–	–	**0.58**									
Co	–	**0.48**	–	**0.71**	**0.78**	–	–								
Cr	0.08	**0.49**	–	0.29	0.26	0.26	–	0.06							
Cu	0.21	**0.54**	**0.30**	**0.37**	**0.59**	–	–	**0.41**	**0.48**						
Hg	–	0.17	0.02	0.24	**0.40**	0.04	–	0.31	0.05	0.11					
Ni	–	–	0.13	–	–	**0.38**	**0.68**	–	–	–	0.10				
Pb	0.06	0.28	**0.39**	0.12	0.35	0.10	–	0.13	0.29	**0.48**	0.17	0.03			
Sb	0.24	**0.46**	–	**0.39**	**0.51**	0.16	–	0.35	**0.54**	0.33	0.34	–	–		
Tl	0.07	–	0.21	–	–	**0.62**	**0.80**	–	–	–	0.13	**0.66**	0.04	0.06	
Zn	0.26	**0.71**	0.17	0.34	**0.44**	–	–	0.22	**0.41**	**0.58**	0.01	–	**0.46**	**0.39**	–

Bold indicates satistically significant at *p* < 0.05; 0 < *r *< 0.3 very low correlation; 0.3 ≤ *r* < 0.5 low correlation; 0.5 ≤ *r* < 0.7 medium correlation; 0.7 ≤ *r* < 0.9 strong correlation; 0.9 ≤ *r* < 1 very strong correlation; –, not applicable

**Table 7:** A component matrix for variables (*n* = 30)

Variables	PCA 1	PCA 2
pH_KCl_	0.205	0.190
Clay	**0.759**	− 0.148
*C*_org_	0.225	0.081
Al	**0.634**	− 0.270
Fe	**0.794**	− 0.140
As	0.069	**0.733**
Cd	0.042	**0.868**
Co	**0.678**	− 0.292
Cr	**0.514**	0.074
Cu	0.497	− 0.041
Hg	0.488	0.201
Ni	− 0.283	**0.811**
Pb	0.311	0.261
Sb	**0.717**	0.312
Tl	− 0.112	**0.882**
Zn	**0.560**	− 0.254
Eigenvalue	4.194	2.987
Total variance %	26.21	18.66
Cumulative variance %	26.21	44.88

Factor loadings exceeding 0.5 are shown in bold

### Human health risk assessment

The values of calculated non-carcinogenic and carcinogenic risk arising from ingestion, dermal contact, and inhalation pathways, under residential, recreational, and agricultural (worker) scenarios are presented in Table 8. Under each exposure scenario, both non-carcinogenic and carcinogenic risk values were higher for children than for adults. The total non-carcinogenic risks were equal to 1.63E−01 for adults and 4.55E−01 for children under the residential scenario; 2.88E−01 for adults and 6.69E−01 for children under the recreational scenario; and 1.03E−01 for adult workers under the agricultural scenario. Therefore, for both adults and children under the three assumed exposure scenarios, the total non-carcinogenic risk values were < 1; thus, adverse health effects were not likely to be observed. The total carcinogenic risks (of carcinogenic PHEs investigated in the study) were equal to 1.62E−05 for adults and 6.39E−05 for children under the residential scenario, 5.41E−06 for adults and 2.46E−05 for children under the recreational scenario, and 1.45E−05 for adult workers under the agricultural scenario. Therefore, for both adults and children under the three exposure scenarios, the total carcinogenic risk values were at the acceptable levels that were set to be equal to 1 × 10^−4^ for the sum of carcinogenic PHEs. Under all the exposure scenarios, non-carcinogenic and carcinogenic risks decreased in the following order of exposure pathways: ingestion > dermal contact > inhalation of soil particles, for both adults and children, with the exception of adults under the residential scenario where the carcinogenic risk decreased as follows: dermal contact > ingestion > inhalation of soil particles. For the inhalation exposure pathway under each scenario and for adults and children, the non-carcinogenic risks decreased as follows: Co > As > Cr(VI) > Ni > Cd > Hg > Se. For the ingestion pathway under the residential scenario for adult and children and under the agricultural scenario for adult workers, non-carcinogenic risks decreased as follows: Pb > Co > As > Tl > Cr(VI) > Sb > Zn > Cu > Ni > Cd > Hg > Se > Cr(III). For the dermal contact by adults and children under the residential scenario, for adults under the recreational scenario, and for adult workers under the agricultural scenario, the decreasing order was the following: Cr(VI) > As > Pb > Sb > Co > Cd > Ni > Tl > Hg > Cr(III) > Zn > Cu > Se. Under the recreational scenario for adults and children in the ingestion pathway and for children in the dermal contact exposure, the decreasing order was the following: As > Pb > Co > Tl > Cr(VI) > Sb > Zn > Cu > Ni > Cd > Hg > Se > Cr(III). For the carcinogenic risk of the inhalation exposure under all the exposure scenarios, the decreasing order of investigated PHEs was as follows: Cr(VI) > Co > As > Ni > Cd, for both adults and children, whereas in the case of ingestion and dermal contact by adults and children, the decreasing order was the following: Cr(VI) > As > Pb.

**Table 8:** Non-carcinogenic and carcinogenic PHE risks in the agricultural soils of southern Poland

Residential								
Ingestion					Dermal contact			
PHEs	HQ adult	HQ child	CR adult	CR child	HQ adult	HQ child	CR adult	CR child
As	1.90E−02	4.43E−02	2.93E−06	1.99E−05	4.01E−03	2.10E−02	6.18E−07	8.13E−07
Cd	5.57E−04	1.30E−03	–	–	9.41E−04	4.94E−03	–	–
Co	2.34E−02	5.47E−02	–	–	9.89E−04	5.19E−03	–	–
Cr (III)	1.79E−05	4.18E−05	–	–	5.82E−05	3.05E−04	–	–
Cr (VI)	8.95E−03	2.09E−02	4.60E−06	3.13E−05	1.51E−02	7.93E−02	7.78E−06	9.93E−06
Cu	9.50E−04	2.22E−03	–	–	4.01E−05	2.10E−04	–	–
Hg	2.38E−04	5.56E−04	–	–	1.44E−04	7.53E−04	–	–
Ni	8.21E−04	1.92E−03	–	–	8.67E−04	4.55E−03	–	–
Pb	6.08E−02	1.42E−01	2.66E−07	1.81E−06	2.57E−03	1.35E−02	1.12E−08	1.50E−08
Sb	4.39E−03	1.03E−02	–	–	1.24E−03	6.49E−03	–	–
Se	2.29E−05	5.33E−05	–	–	3.22E−06	1.69E−05	–	–
Tl	1.43E−02	3.33E−02	–	–	6.03E−04	3.16E−03	–	–
Zn	1.35E−03	3.14E−03	–	–	5.69E−05	2.98E−04	–	–
HI_PHE_/CR_PHE_	1.35E−01	3.14E−01	7.80E−06	5.31E−05	2.66E−02	1.40E−01	8.41E−06	1.08E−05

Residential								
Inhalation					HIt		CRt	
PHEs	HQ adult	HQ child	CR adult	CR child	Adult	Child	Adult	Child
As	3.25E−04	3.25E−04	7.20E−15	1.80E−15	1.63E−01	4.55E−01	1.62E−05	6.39E−05
Cd	2.87E−05	2.87E−05	1.77E−16	4.42E−17				
Co	6.03E−04	6.03E−04	1.12E−14	2.79E−15				
Cr (III)	–	–	–	–				
Cr (VI)	1.38E−04	1.38E−04	3.98E−13	9.65E−14				
Cu	–	–	–	–				
Hg	1.23E−07	1.23E−07	–	–				
Ni	9.40E−05	9.40E−05	7.54E−16	1.88E−16				
Pb	–	–	–	–				
Sb	–	–	–	–				
Se	2.94E−09	2.94E−09	–	–				
Tl	–	–	–	–				
Zn	–	–	–	–				
HI_PHE_/CR_PHE_	1.19E−03	1.19E−03	4.17E−13	1.04E−13				

Recreational								
Ingestion					Dermal contact			
PHEs	HQ adult	HQ child	CR adult	CR child	HQ adult	HQ child	CR adult	CR child
As	2.34E−01	5.46E−01	1.20E−06	8.19E−06	1.05E−03	5.53E−03	1.63E−07	2.14E−07
Cd	2.29E−04	5.34E−04	–	–	2.47E−04	3.25E−05	–	–
Co	9.63E−03	2.25E−02	–	–	2.60E−04	1.36E−03	–	–
Cr (III)	7.36E−06	1.72E−05	–	–	1.53E−05	1.04E−06	–	–
Cr (VI)	3.68E−03	8.58E−03	1.89E−06	1.29E−05	3.98E−03	5.21E−04	2.05E−06	2.61E−06
Cu	3.90E−04	9.11E−04	–	–	1.06E−05	5.53E−05	–	–
Hg	9.78E−05	2.28E−04	–	–	3.78E−05	1.39E−05	–	–
Ni	3.38E−04	7.88E−04	–	–	2.28E−04	4.79E−05	–	–
Pb	2.50E−02	5.83E−02	1.09E−07	7.43E−07	6.75E−04	3.54E−03	2.95E−09	3.95E−09
Sb	1.81E−03	4.21E−03	–	–	3.25E−04	2.56E−04	–	–
Se	9.39E−06	2.19E−05	–	–	8.46E−07	1.33E−06	–	–
Tl	5.87E−03	1.37E−02	–	–	1.59E−04	8.32E−04	–	–
Zn	5.54E−04	1.29E−03	–	–	1.50E−05	7.85E−05	–	–
HI_PHE_/CR_PHE_	2.81E−01	6.57E−01	3.20E−06	2.18E−05	7.00E−03	1.23E−02	2.21E−06	2.83E−06

Recreational								
Inhalation					HIt		CRt	
PHEs	HQ adult	HQ child	CR adult	CR child	Adult	Child	Adult	Child
As	1.43E−05	1.43E−05	3.16E−16	7.89E−17	2.88E−01	6.69E−01	5.41E−06	2.46E−05
Cd	1.26E−06	1.26E−06	7.76E−18	1.94E−18				
Co	2.64E−05	2.64E−05	4.89E−16	1.22E−16				
Cr (III)	–	–	–	–				
Cr (VI)	6.06E−06	6.06E−06	1.75E−14	4.36E−15				
Cu	–	–	–	–				
Hg	5.37E−09	5.37E−09	–	–				
Ni	4.12E−06	4.12E−06	3.30E−17	8.26E−18				
Pb	–	–	–	–				
Sb	–	–	–	–				
Se	1.29E−10	1.29E−10	–	–				
Tl	–	–	–	–				
Zn	–	–	–	–				
HI_PHE_/CR_PHE_	5.21E−05	5.21E−05	1.83E−14	4.57E−15				

Agricultural (worker)								
Ingestion			Dermal contact		Inhalation		HIt	CRt
PHEs	HQ adult	CR adult	HQ adult	CR adult	HQ adult	CR adult	Adult	Adult
As	1.30E−02	3.34E−06	1.60E−03	4.12E−07	7.43E−05	2.74E−15	1.03E−01	1.45E−05
Cd	3.82E−04	–	3.77E−04	–	6.55E−06	6.73E−17		
Co	1.60E−02	–	3.96E−04	–	1.38E−04	4.25E−15		
Cr (III)	1.23E−05	–	2.33E−05	–		–		
Cr (VI)	6.13E−03	5.26E−06	6.06E−03	5.19E−06	3.16E−05	1.51E−13		
Cu	6.51E−04	–	1.61E−05	–	–	–		
Hg	1.63E−04	–	5.75E−05	–	2.80E−08	–		
Ni	5.63E−04	–	3.47E−04	–	2.15E−05	2.87E−16		
Pb	4.16E−02	3.03E−07	1.03E−03	7.49E−09	–	–		
Sb	3.01E−03	–	4.95E−04	–		–		
Se	1.57E−05	–	1.29E−06	–	6.72E−10	–		
Tl	9.78E−03	–	2.42E−04	–	–	–		
Zn	9.23E−04	–	2.28E−05	–	–	–		
HI_PHE_/CR_PHE_	9.23E−02	8.90E−06	1.07E−02	5.61E−06	2.72E−04	1.59E−13		

–, not applicable

### Uncertainties in HHRA

The calculated HHRA values were assumed due to the fact that exposure parameters were equivalent to either default data or certain assumptions adopted for research purposes. Thus, especially in the case of the recreational scenario, the risk values depended strongly on real exposition. In the calculations, the RBA and GIABS factors were used to determine bioavailable contents of PHEs (see Table 2); however, such factors were not available for all PHEs; thus, the risk values may have been overestimated. Only twelve PHEs as non-carcinogenic were analyzed and, out of them, only five in the inhalational pathway and three in ingestion and dermal contact were considered to be carcinogenic; thus, the real total non-carcinogenic and carcinogenic risks may have been underestimated. Under the residential scenario, where the highest carcinogenic risk in the dermal contact pathway was observed, the risk may have been caused by the assumption of the total Cr content in soil ascribed to Cr(VI) and that obviously caused risk value overestimation.

## Discussion

The research on agricultural soils in southern Poland was performed in 2015 and 2016, in the areas where edible plants had been cultivated and subsequently sold on fresh produce markets by the farmers. The concentration of neither of the investigated PHEs (As, Cd, Cr, Co, Cu, Hg, Ni, Pb, Sb, Se, Tl, and Zn) exceeded permissible concentrations, in reference to either the Polish law standards or the Canadian guidelines for the protection of environmental and human health. Thus, the investigated soils met the legal requirements for the cultivation of edible plants under the Polish law and there was no threat to human health or the environment, in reference to the applicable soil guidelines. The results were in accordance with the findings of the last 20-year National Monitoring System of Arable Soils program in Poland that revealed that the PHE contents in the majority of arable soils were low and do not adversely affect soil functions for high-quality food production (Smreczak et al. 2018). The mean Al concentration was equal to 0.65% and that of Fe to 0.98%. The mean PHE contents were the following (mg/kg): As 6.64, Cd 0.39, Co 4.92, Cr 18.8, Cu 26.5, Hg 0.05, Ni 11.5, Pb 63.8, Sb 1.23, Tl 0.10, Zn 283, and Se below the limit of quantification. The results obtained in the current confirmed the findings of other researchers on the PHE contents in the arable soils of southern Poland (Loska et al. 2003, 2004; Baran and Wieczorek 2015; Mazurek et al. 2017; Labaz et al. 2019; Baran et al. 2018; Gałuszka et al. 2018; Pająk et al. 2018; Piekut et al. 2018; Wieczorek et al. 2018; Mazurek et al. 2019; Waroszewski et al. 2019). Nevertheless, the direct comparison of data between different studies should be made with caution due to time and methods differences.

The bioavailable element contents of PHEs, extracted in CaCl_2_, were the following: Cd 15.4%, Cr 0.03%, Cu 0.19%, Fe 0.01%, Ni 0.96%, Pb 0.11%, and Zn 0.48% of the total PHE content. Taking under consideration low extraction efficiency of CaCl_2_, which, however, indicated significant mobility of cadmium (Ali et al. 2018), further more detailed study on PHE mobility and availability of investigated arable soils seems to be justified.

Quartz was the main mineral found in the investigated soils, followed by small amounts of feldspars, illite, montmorillonite, kaolinite, and goethite. Due to a very similar mineral composition of the investigated soil samples, one could conclude that the differences identified in the PHE contents could be associated with the anthropogenic activities rather than the mineral composition of the investigated soils. High positive (Al, Fe, Co, Cu, Cr, Sb, and Zn) and negative (Ni) correlations between the elements and the clay content may have indicated that the clay fraction played a dominant role in the PHE mobility and sorption. The positive correlations observed might also indicate the origin of Co, Cu, Hg, Sb, Zn, Cr, and Pb from natural sources in the investigated agricultural soils. On the other hand, the negative correlations between some individual pairs of trace elements might point at the anthropogenic origin of Cd, Ni, As, and Tl in agricultural soils.

As mentioned above the majority of arable soils in Poland were not contaminated with PHEs according to legal requirements. However, locally or even regionally there might be areas where exceedance of PHEs content might occur. Then ecological and health risk assessment procedures should be applied. The *I*_geo_ classification indicated a moderate contamination of soils by Zn, uncontaminated to moderate contamination by Pb, and no contamination by other investigated PHEs. The EF factor revealed a moderate severe enrichment with Zn, moderate enrichment with Pb and Se, minor enrichment with As, Cd, Cu, and Hg, and no enrichment with Co, Cr, Ni, Sb, or Tl. The CF factor pointed at a considerable contamination by Pb and Zn, moderate contamination by As, Cd, Cu, Hg, and Sb, and low contamination by Co, Cr, Ni, Se, and Tl. The PI index indicated a strong pollution by Zn, moderate pollution by As, Cd, Cu, Pb, and Se, and lack of pollution by Co, Cr, Hg, Ni, Sb, or Tl. Finally, the PI_T_ index indicated a lack of pollution by each PHE. The Er coefficient indicated a considerable potential ecological risk of Cd, moderate potential ecological risk of Hg, and low potential ecological risk of As, Co, Cr, Cu, Ni, Pb, Sb, Tl, and Zn. The mHQ values revealed moderate severity of contamination by Zn, low severity by Pb, very low severity by As, Cu, and Ni, and none to very low severity by Cd, Cr, or Hg. The values of the mPELq quotient indicated a medium–low degree of contamination and the mERMq quotient a medium–low priority site.

The value of the ECI index classified the analyzed soils at the border between the uncontaminated and uncontaminated to slightly contaminated. The CSI index value indicated that the studied soils were not contaminated by the investigated PHEs in the southern Poland.

The total non-carcinogenic risks were equal to 1.63E−01 for adults and 4.55E−01 for children under the residential scenario, 2.88E−01 for adults and 6.69E−01 for children under the recreational scenario, and 1.03E−01 for adult workers under the agricultural scenario. For both adults and children under the three assumed exposure scenarios, the total non-carcinogenic risk values were < 1; thus, adverse health effects were not likely to be observed. The total carcinogenic risk of As, Cr(VI), and Pb in ingestion and dermal contact exposure and of As, Cd, Co, Cr(VI), and Ni in inhalation exposure was equal to 1.62E−05 for adults and 6.39E−05 for children under the residential scenario, 5.41E−06 for adults and 2.46E−05 for children under the recreational scenario, and 1.45E−05 for adult workers under the agricultural scenario. For both adults and children under the three exposure scenarios, the total carcinogenic risk values were at the acceptable levels that were set to be equal to 1 × 10^−4^ for the sum of carcinogenic PHEs.

## Conclusions

The studies revealed that investigated agricultural soils were fully suitable for edible plants production. The permissible total PHE concentrations were not exceeded. Despite the increasing environmental pollution with PHEs, especially in Śląskie and Małopolskie Regions of the southern Poland, the quality of the arable soils was not lowered due to several reasons. The soils in Poland were generally not significantly contaminated, although it should be noted that there were point pollution sources of soils with PHEs, what was observed even in the investigated area. Moreover, it is worth remembering that agricultural soils were intensively used for the plant production; thus, some of investigated elements were taken up by plants and removed at once with the crop yield. Thus, agricultural soils should remain under special supervision to maintain their quality for food production. Finally, most of analyzed PHEs indicated low mobility due to properties of the studied soils. However, the next step of the research should be the analysis of the PHE bioavailability of these particular soils. To sum up, the study of total and soluble forms of PHE contents in the arable soils, with the ecological and health risk implications, was an important step in determining the potential transfer of PHEs in the soil–plant–human chain. The applied soil quality and contamination indices managed to reveal the PHEs content in soils in various conditions. The achieved results could be useful in planning, risk assessment, and decision making in the environmental management and for improving the ecosystem and human health.

## Electronic supplementary material

Below is the link to the electronic supplementary material.

Supplementary material 1 (DOCX 44 kb)
