# Supplementary material for: The contents of the potentially harmful elements in the arable soils of southern Poland, with the assessment of ecological and health risks: a case study
Source: Environ Geochem Health. 2019 Jul 19;42(2):419–42. doi: 10.1007/s10653-019-00372-w (PMC7035217; doi:10.1007/s10653-019-00372-w)
Supplement: Supplementary file 1 — Supplementary material 1 (DOCX 44 kb) [file 10653_2019_372_MOESM1_ESM.docx]

**The contents of the potentially harmful elements in the arable soils of southern Poland, with the assessment of ecological and health risks: a case study**

**Environmental Geochemistry and Health**

**Electronic Supplementary Material**

Agnieszka Gruszecka-Kosowska^1*^, Agnieszka Baran^2^, Magdalena Wdowin^3^, Katarzyna Mazur-Kajta^4^, Tomasz Czech^2^

^1^AGH University of Science and Technology, Faculty of Geology, Geophysics, and Environmental Protection, Department of Environmental Protection, al. Mickiewicza 30, 30-059 Kraków, Poland

E-mail: agnieszka.gruszecka@agh.edu.pl, Phone: +48 12 617 50 33, Fax: +48 12 633 29 36

^*^Corresponding author

ORCID: Agnieszka Gruszecka-Kosowska 0000-0002-4988-173X

^2^University of Agriculture in Krakow, Faculty of Agriculture and Economics, Department of Agricultural and Environmental Chemistry, al. Mickiewicza 21, 31-120 Kraków, Poland

ORCID: Agnieszka Baran 0000-0003-4697-2959

^3^Magdalena Wdowin, Mineral and Energy Economy Research Institute, Polish Academy of Sciences, ul. J. Wybickiego 7A, 31-261 Kraków, Poland

ORCID: Magdalena Wdowin 0000-0002-5097-719X

^4^Opole University of Technology, Faculty of Economics and Management, Department of International Economic Relations, ul, Prószkowska 76, 45-758 Opole, Poland

ORCID: Katarzyna Mazur-Kajta 0000-0002-4822-9328

**Electronic Supplementary Material captions**

**ESM_1**. Descriptions of the single and complex soil contamination indices used in the study

**ESM_2**. Descriptions of the single and complex ecological risk indices used in the study

**ESM_1**. Descriptions of the single and complex soil contamination indices used in the study

| **Indices** | **Formulas** | **Explanations** | **Limit values** | **Classification** | **References** |
| --- | --- | --- | --- | --- | --- |
| **Single indices** | | | | | |
| Geoaccumulation index  I_geo_ | I_geo_=log_2_*(C_i_/1.5*B_n_) | C_i_ – content of single PHE in soil; B_n_ –background value; 1.5 – constant | I_geo_ ≤0 | Class 0 - practically uncontaminated | Müller 1969; Kowalska et al. 2018 |
|  |  |  | 0≤ I_geo_<1 | Class I - uncontaminated to moderately contaminated |  |
|  |  |  | 1≤ I_geo_<2 | Class II - moderately contaminated |  |
|  |  |  | 2≤ I_geo_<3 | Class III - moderately to heavily contaminated |  |
|  |  |  | 3≤ I_geo_<4 | Class IV - heavily contaminated |  |
|  |  |  | 4≤ I_geo_<5 | Class V - heavily to extremely contaminated |  |
|  |  |  | 5≤ Igeo | Class VI - extremely contaminated |  |
| Enrichment factor  EF | EF=(C_i_/C_ref_)/(B_i_/B_ref_) | C_i_ – content of single PHE in soil; C_ref_ – content of Fe in sample; B_i_ – reference content of single PHE; B_ref_ – reference content of Fe | EF ≤1 | no enrichment | Sutherland 2000;  Ho et al. 2010 |
|  |  |  | 1< EF ≤3 | minor enrichment |  |
|  |  |  | 3< EF ≤5 | moderate enrichment |  |
|  |  |  | 5< EF ≤10 | moderately severe enrichment |  |
|  |  |  | 10< EF ≤25 | severe enrichment |  |
|  |  |  | 25< EF ≤50 | very severe enrichment |  |
|  |  |  | EF >50 | extremely severe enrichment |  |
| Contamination factor  CF | CF = Cm_i_/C_ref_ | Cm_i_ – mean PHE concentration from at least five soil samples; C_ref_ – reference value of PHE | CF <1 | low contamination factor | Håkanson 1980; Loska et al. 2004 |
|  |  |  | 1≤ CF <3 | moderate contamination factor |  |
|  |  |  | 3≤ CF <6 | considerable contamination factor |  |
|  |  |  | 6 ≤CF | very high contamination factor |  |
| Single pollution index  PI | PI = C_i_/B_i_ | C_i_ – content of single PHE in soil; B_i_ - background value | PI <1 | unpolluted, low level of pollution | Weissmanová and Pavlovský 2017 |
|  |  |  | 1≤ PI <3 | moderately polluted |  |
|  |  |  | 3 ≤PI | strongly polluted |  |
| Threshold pollution index PI_T_ | PI_T_ = C_i_/Si | C_PHE_ – content of single PHE in soil; Si - standard value | PI_T_ <1 | unpolluted | Weissmanová and Pavlovský 2017 |
|  |  |  | 1≤ PI_T_ <2 | low pollution |  |
|  |  |  | 2≤ PI_T_ <3 | moderate pollution |  |
|  |  |  | 3≤ PI_T_ <5 | strong pollution |  |
|  |  |  | 5≤ PI_T_ | very strong pollution |  |
| Potential contamination index  PCI | PCI = Ci_max_/ Bi | Ci – max content of single PHE in soil; Bi - background value | PCI <1 | low contamination | Benson et al. 2018 |
|  |  |  | 1< PCI <3 | moderate contamination |  |
|  |  |  | PCI >3 | severe contamination |  |
| **Indices** | **Formulas** | **Explanations** | **Limit values** | **Classification** | **References** |
| **Complex indices** | | | | | |
| Contamination degree  C_deg_ | C_deg_ = $\sum_{i=1}^{n} \mathrm{CF}$ | CF – contamination factor for single PHE; n – number of PHEs | C_deg_ <8 | low degree of contamination | Loska et al. 2004 |
|  |  |  | 8≤ C_deg_ <16 | moderate degree of contamination |  |
|  |  |  | 16≤ C_deg_ <32 | considerable degree of contamination |  |
|  |  |  | 32≤ C_deg_ | very high degree of contamination |  |
| Modified contamination degree  mC_deg_ | mC_deg_ = $\frac{\sum_{i=1}^{n} \mathrm{CF}}{n}$ | CF – contamination factor for single PHE; n – number of PHEs | mC_deg_ <1.5 | nil to very low degree of contamination | Abrahim and Parker 2008 |
|  |  |  | 1.5≤mC_deg_<2 | low degree of contamination |  |
|  |  |  | 2≤ mC_deg_ <4 | moderate degree of contamination |  |
|  |  |  | 4≤ mC_deg_ <8 | high degree of contamination |  |
|  |  |  | 8≤ mC_deg_ <16 | very high degree of contamination |  |
|  |  |  | 16≤ mC_deg_ <32 | extremely high degree of contamination |  |
|  |  |  | 32≤ mC_deg_ | ultra high degree of contamination |  |
| Sum of pollution index  PI_sum_ | PI_sum_ =$\sum_{i=1}^{n} \mathrm{PI}$ | PI – pollution index for single PHE; n – number of PHEs | no limit values | sum of PHE contamination in soil | Kowalska et al. 2016;  Gong et al. 2008 |
| Sum of threshold pollution index  PI_Tsum_ | PI_Tsum_ =$\sum_{i=1}^{n} \mathrm{PI}$ | PI_T_ – threshold pollution index for single PHE; n – number of PHEs | no limit values | sum of PHE contamination in soil | Kowalska et al. 2016;  Gong et al. 2008 |
| Average pollution index  PI_Avg_ | PI_Avg_ = $\frac{1}{n}$ $\sum_{i=1}^{n} \mathrm{PI}$ | PI – single PI for analyzed PHEs; n – number of PHEs | PI_Avg_ >1 | low quality of soil because of contamination | Gong et al. 2008 |
| Average pollution index  PI_TAvg_ | PI_TAvg_ = $\frac{1}{n}$ $\sum_{i=1}^{n} \mathrm{PI}$ | PI_T_ – single PI for analyzed PHEs; n – number of PHEs | PI_TAvg_ >1 | low quality of soil because of contamination | Gong et al. 2008 |
| Pollution load index  PLI | PLI = $\sqrt[n]{\mathrm{PI}1 x PI2 x \ldots x PIn}$ | PI – pollution index for single PHE; n – number of PHEs | PLI <1 | unpolluted | Weissmanová and Pavlovský 2017 |
|  |  |  | PLI =1 | baseline level of pollution |  |
|  |  |  | PLI >1 | polluted |  |
| Improved Nemerow pollution index  PI_N_ | PI_N_ = $\sqrt{\frac{{(\frac{1}{n} \sum_{i=1}^{n} \mathrm{PI})}^{2}+ \mathrm{PImax}^{2}}{n}}$ | PI – pollution index for single PHE; max PI of all PHEs; n – number of PHEs | PI_N_ <0.7 | Grade 1- safety domain | Cheng et al. 2007 |
|  |  |  | 0.7≤ PI_N_ <1.0 | Grade 2 - precaution domain |  |
|  |  |  | 1.0≤ PI_N_ <2.0 | Grade 3 - slightly polluted domain |  |
|  |  |  | 2.0≤ PI_N_ <3.0 | Grade 4 - moderately polluted domain |  |
|  |  |  | PI_N_ >3.0 | Grade 5 - seriously polluted domain |  |

**ESM_2.** Descriptions of the single and complex ecological risk indices used in the study

| **Indices** | **Formulas** | **Explanations** | **Limit values** | **Classification** | **References** |
| --- | --- | --- | --- | --- | --- |
| **Single indices** | | | | | |
| Potential ecological risk coefficient  Er | Er = Tr_i_ x CF_i_ | Tr – toxicity response coefficient of single PHE; CF_i_ – contamination factor of single PHE | Er <40 | low potential ecological risk | Håkanson 1980;  Gong et al. 2008;  Chai et al. 2017;  Liu et al. 2018;  Wang et al. 2018 |
|  |  |  | 40≤ Er <80 | moderate potential ecological risk |  |
|  |  |  | 80 ≤Er <160 | considerable potential ecological risk |  |
|  |  |  | 160≤ Er <320 | high potential ecological risk |  |
|  |  |  | 320≤ Er | very high potential ecological risk |  |
| Hazard quotient  HQ | HQ = C_i_ / S_i_ | Ci – content of single PHE in soil; Si – standard value | HQ <0.1 | no adverse effects | Swartjes et al. 2008; Feng et al. 2011 |
|  |  |  | 0.1< HQ <1 | potential hazard |  |
|  |  |  | 1< HQ <10 | moderate hazard |  |
|  |  |  | HQ >10 | high hazard |  |
| Modified hazard quotient  mHQ | mHQ = [Ci ($\frac{1}{\mathrm{TELi}}$ + $\frac{1}{\mathrm{PELi}}+ \frac{1}{\mathrm{SELi}}$)]${\begin{aligned} \\ \end{aligned}}^{2}$ | Ci – concentration of PHE in soil; TEL – threshold effect level; PEL – probable effect level; SEL – severe effect level for single PHEs | mHQ <0.5 | nil to very low severity of contamination | Benson et al. 2018; MacDonald et al. 2000 |
|  |  |  | 0.5< mHQ <1.0 | very low severity of contamination |  |
|  |  |  | 1.0< mHQ <1.5 | low severity of contamination |  |
|  |  |  | 1.5< mHQ <2.0 | moderate severity of contamination |  |
|  |  |  | 2.0< mHQ <2.5 | considerable severity of contamination |  |
|  |  |  | 2.5< mHQ <3.0 | high severity of contamination |  |
|  |  |  | 3.0< mHQ <3.5 | very high severity of contamination |  |
|  |  |  | mHQ >3.5 | extreme severity of contamination |  |
| **Complex indices** | | | | | |
| Potential ecological risk index  RI | RI = $\sum_{i=1}^{n} \mathrm{Eri}$ | Eri – potential ecological risk coefficient for single PHE; n – number of analyzed PHEs | RI <150 | low ecological risk | Inengite et al. 2015; Weissmanová and Pavlovský 2017 |
|  |  |  | 150≤ RI <300 | moderate ecological risk |  |
|  |  |  | 300≤ RI <600 | considerate ecological risk |  |
|  |  |  | RI >600 | very high ecological risk |  |
| **Indices** | **Formulas** | **Explanations** | **Limit values** | **Classification** | **References** |
| Hazard index  HI | HI = $\sum_{i=1}^{n} \mathrm{HQi}$ | HQi – hazard quotient for single PHE;  n – number of analyzed PHEs | HQ <0.1 | no adverse effects | Swartjes et al. 2008; Feng et al. 2011 |
|  |  |  | 0.1< HQ <1 | potential hazard |  |
|  |  |  | 1< HQ <10 | moderate hazard |  |
|  |  |  | HQ >10 | high hazard |  |
| Mean probable effect level quotient  mPELq | mPEL_Q_ = $\frac{\sum_{i=1}^{n} (\frac{\mathrm{Ci}}{\mathrm{PELi}})}{n}$ | Ci – concentration of PHE in soil; PELi – probable effect level for single PHE; n – sum of PHEs considered | ≤ 0.1 | low degree of contamination: 8% probability of being toxic | Benson et al. 2018; Long et al. 2006 |
|  |  |  | 0.11–1.5 | medium-low degree of contamination: 21% probability of being toxic |  |
|  |  |  | 1.51–2.3 | high-medium degree of contamination: 49% probability of being toxic |  |
|  |  |  | > 2.3 | high degree of contamination: 73% probability of being toxic |  |
| Mean effect range median quotient mERMq | mERM_Q_ = $\frac{\sum_{i=1}^{n} (\frac{\mathrm{Ci}}{\mathrm{ERMi}})}{n}$ | Ci – concentration of PHE in soil; ERMi –effect range median for single PHE; n – sum of PHEs considered | ≤ 0.1 | low priority site: 9% probability of being toxic | Benson et al. 2018; Long et al. 2000 |
|  |  |  | 0.1–0.5 | medium-low priority site: 21% probability of being toxic |  |
|  |  |  | 0.5–1.5 | high-medium priority site: 49% probability of being toxic |  |
|  |  |  | > 1.5 | high priority site: 76% probability of being toxic |  |
| Ecological contamination index  ECI | ECI = Bn $\sum_{i=1}^{n} \mathrm{mHQi}$ | Bn – reciprocal value of derived eigenvalue of PHE  HQi – hazard quotient for single PHE;  n – number of analyzed PHEs | ECI <2 | uncontaminated | Benson et al. 2018 |
|  |  |  | 2< ECI <3 | uncontaminated to slightly contaminated |  |
|  |  |  | 3< ECI <4 | slightly to moderately contaminated |  |
|  |  |  | 4< ECI <5 | moderately to considerably contaminated |  |
|  |  |  | 5< ECI <6 | considerably to highly contaminated |  |
|  |  |  | 6< ECI <7 | highly contaminated |  |
|  |  |  | ECI >7 | extremely contaminated |  |
| **Indices** | **Formulas** | **Explanations** | **Limit values** | **Classification** | **References** |
| Contamination severity index  CSI | CSI = $\sum_{i=1}^{n} Wt {[(\frac{Ci}{ERLi})}^{\frac{1}{2}}+{[(\frac{Ci}{ERMi})}^{2}]$  Wt = $\frac{Lfi x Ev}{\sum_{i=1}^{n} (Lfi x Ev)}$ | Wt – weighted value for n number of PHEs; Lfi – factor loading for single PHE; Ev – eigenvalue; Ci - content of single PHE in soil; ERLi – effect range low; ERMi – effect range median | CSI <0.5 | uncontaminated | Pejman et al. 2015; Benson et al. 2018 |
|  |  |  | 0.5≤ CSI <1 | very low severity of contamination |  |
|  |  |  | 1≤ CSI <1.5 | low severity of contamination |  |
|  |  |  | 1.5≤ CSI <2 | low to moderate severity of contamination |  |
|  |  |  | 2≤ CSI <2.5 | moderate severity of contamination |  |
|  |  |  | 2.5≤ CSI <3 | moderate to high severity of contamination |  |
|  |  |  | 3≤ CSI <4 | high severity of contamination |  |
|  |  |  | 4≤ CSI <5 | very high severity of contamination |  |
|  |  |  | CSI ≥5 | ultra high severity of contamination |  |

**References**

Abrahim, G.M.S., & Parker, R.J. (2008). Assessment of heavy metal enrichment factors and the degree of contamination in marine sediments from Tamaki Estuary, Auckland, New Zealand. *Environmental Monitoring and Assessment*, 136, 227–238.

Benson, N.U., Adedapo, A.E., Omowunmi, H.F.A., Williams, A.B., Udosen, E.D., Ayejuyo, O.O., & Olajire, A.A. (2018). New ecological risk indices for evaluating heavy metals contamination in aquatic sediment: A case study of the Gulf of Guinea. *Regional Studies in Marine Science*, 18, 44–56.

Chai, L., Li, H., Yang, Z., Min, X., Liao, Q., Liu, Y., Men, S., Yan, Y., & Xu, J. (2017). Heavy metals and metalloids in the surface sediments of the Xiangjiang River, Hunan, China: distribution, contamination, and ecological risk assessment. *Environmental Science and Pollution Research*, 24, 874–885.

Cheng, J-L., Shi, Z., & Zhu, Y-W. (2007). Assessment of mapping of environmental quality of agricultural soils of Zhejiang Province, China. *Journal of Environmental Sciences* 19, 50–54.

Feng, H., Jiang, H., Gao, W., Weinstein, M.P., Zhang, Q., Zhang, W., Yu, L., Yuan, D., & Tao, J. (2011). Metal contamination in sediments of the western Bohai Bay and adjacent estuaries, China. *Journal of Environmental Management*, 92, 1185–1197.

Gong, Q., & Deng, J. (2008). Calculating pollution indices by heavy metals in ecological geochemistry assessment and as case study in parks of Beijing. *Journal of China University of Geosciences*, 19(3), 230–241.

Håkanson, L. (1980). An ecological risk index for aquatic pollution control. A sedimentological approach. *Water Research,* 14, 975–1001.

Ho, H.H., Swennen, R., & Van Damme, A. (2010). Distribution and contamination status of heavy metals in estuarine sediments near Cua Ong Harbo, Ha Long Bay, Vietnam. *Geologica Belgica*, 13(1-2), 37–47.

Inengite, A.K., Abasi, C.Y., & Walter, C. (2015). Application of pollution indices for the assessment of heavy metal pollution in flood impacted soil. *International Research Journal of Pure & Applied Chemistry,* 8(3), 175–189.

Kowalska, J., Mazurek, R., Gąsiorek, M., Setlak, M., Zaleski, T., & Waroszewski, J. (2016). Soil pollution indices conditioned by medieval metallurgical activity – A case study from Krakow (Poland). *Environmental Pollution*, 218, 1023–1036.

Kowalska, J.B., Mazurek, R., Gąsiorek, M., & Zaleski, T. (2018). Pollution indices as useful tools for the comprehensive evaluation of the degree of soil contamination. *Environmental Geochemistry and Health*, 40, 2395–2420.

Liu, Y., Wang, Q., Zhuang, W., Yuan, Y., Jiao, K., Wang, M., & Chen, Q. (2018). Calculation of thallium’s toxicity coefficient in the evaluation of potential ecological risk index: a case study. *Chemosphere*, 194, 562–569.

Long, E.R., Ingersoll, C.G., & MacDonald, D.D. (2000). Classifying probabilities of acute toxicity in marine sediments with empirically derived sediment quality guidelines. *Environmental Toxicology and Chemistry*, 19(10), 2598–2601.

Long, E.R., Ingersoll, C.G., & MacDonald, D.D. (2006). Calculation and uses of mean sediment quality quotients: a critical review. *Environmental Science and Technology*, 40(6), 1726–1736.

Loska, K., Wiechuła, D., & Korus, I. (2004). Metal contamination of farming soils affected by industry. *Environment International,* 30, 159–165.

MacDonald, D.D., Ingersoll, C.G., & Berger, T.A. (2000). Development and evaluation of consensus-based sediment quality guidelines for freshwater ecosystems. *Archives of Environmental Contamination and Toxicology,* 39, 20–31.

Müller, G. (1969). Index of geoaccumulation in sediments of the Rhine River, *GeoJournal,* 2(3), 108–118.

Pejman, A., Bidhendi, G.B., Ardestani, M., Saeedi, M., & Baghvand, A. (2015). A new index for assessing heavy metals contamination in sediments: A case study. *Ecological Indicators*, 58, 365–373.

Sutherland, R.A. (2000). Bed sediment-associated trace metals in an urban stream, Oahu, Hawaii. *Environmental Geology*, 39(6), 611–627.

Swartjes, F.A., Carlon, C., Niek, H.S.M., & de Wit, N.H.S.M. (2008). The possibilities for the EU-wide use of similar ecological risk-based soil contamination assessment tools. *Science of the Total Environment*, 406, 523–529.

Wang, N., Wang, A., Kong, L., & He, M. (2018). Calculation and application of Sb toxicity coefficient for potential ecological risk assessment. *Science of the Total Environment*, 610–611, 167–174.

Weissmanová, H.D., & Pavlovský, J. (2017). Indices of soil contamination by heavy metals – methodology of calculation for pollution assessment (minireview). *Environmental Monitoring and Assessment*, 189(16):616.
